# Sustainable Supercritical Carbon Dioxide Extraction of Value-Added Lignan from Sesame Meal: Achieving Green Neuroprotection and Waste Valorization by Optimizing Temperature, Solvent, and Pressure

**DOI:** 10.3390/molecules30030539

**Published:** 2025-01-24

**Authors:** Kuo-Ching Jan, Mohsen Gavahian

**Affiliations:** Department of Food Science, National Pingtung University of Science and Technology, No. 1, Xuefu Rd., Neipu, Pingtung 91201, Taiwan

**Keywords:** food processing by-product, emerging food processing technologies, non-thermal technologies, waste valorization, neurological treatment, lignan, sesame oil cake, sustainability

## Abstract

In pursuing sustainable health solutions and growing demand for neuroprotective interventions, the industry demands alternative green extraction technologies to valorize agri-food by-products. This study aimed to develop an optimized supercritical carbon dioxide extraction to isolate sesame meal’s functional compound (lignans) and assess their neuroprotective effects. Extraction was performed at various pressures (2–4 kpsi), temperatures (40–60 °C), co-solvent concentrations (2–25 mol% ethanol), and CO_2_ collection segments (0–100 NL) to systematically analyze extraction parameters. Extracts were analyzed quantitatively using high-performance liquid chromatography followed by neuroprotective mechanisms analysis through PC12 neural cell and ischemic stroke models. The results showed that adding ethanol enhanced the polarity and density of supercritical CO_2_, improving the extraction efficiency of polar lignans. Optimal extraction conditions (4 kpsi, 50 °C, 10 mol% ethanol) yielded the highest sesamol, sesamin, and sesamolin. Extracts showed remarkable protective capabilities when subjected to oxygen–glucose deprivation (OGD) conditions simulating ischemic stress, preventing the enhancement of lactate dehydrogenase activity. Relatively low extract concentrations (25–100 μg/mL) significantly mitigated cellular damage induced by short and extended OGD conditions. The findings revealed green extraction methodologies’ capability to transform sesame meal, a food processing waste, into value-added compounds, in line with sustainable development goals for responsible and sustainable food production, particularly SDGs 3, 9, 12, and 13.

## 1. Introduction

Natural plants and their derivatives continue to serve as a primary source of bioactive compounds in food and therapeutics, with sesame being a particularly notable example. Among the various bioactive constituents found in sesame, lignans represent a significant class of compounds with diverse chemical structures and potential health benefits. The primary lignans in sesame include sesamin, sesamolin, sesamol, sesamol dimer, sesamolinol, sesaminol, and pinoresinol. Sesamin and sesamolin are the most abundant, constituting 0.2–0.5% and 0.1–0.3% of sesame seeds, respectively. These low-molecular-weight compounds are formed through the oxidative coupling of two p-hydroxyphenyl propane molecules and are widely distributed in plants [1].

The lignan content of sesame seeds exhibits remarkable variation across different varieties and seed types, with white and black sesame seeds demonstrating distinct sesamin profiles that positively correlate with their oil content. Research has revealed substantial biochemical diversity among black sesame varieties, where some species accumulate high levels of specific lignans while others develop unique compound configurations. Sesame lignans are particularly intriguing due to their complex chemical transformations, as compounds such as sesamin and sesamolin initially lack direct antioxidant activity but serve as critical precursors for phenolic compounds such as sesamin, sesamolin, sesamol, sesamol dimer, sesamolinol, sesaminol, and pinoresinol. These lignans undergo significant molecular modifications during processing stages such as oil refining, heating, and deodorization, generating novel chemical structures with potential biological activities and therapeutic implications [2]. Lignan glucosides represent a critical component of sesame’s biochemical profile. In white sesame seeds, lignan glucosides typically range from 100 to 170 mg/g of seed, with sesaminol triglucoside being the most abundant, accounting for 80–90% of the total lignan glucoside content. Interestingly, seed germination processes can alter the composition of these compounds, with sesamin and sesamolin gradually diminishing while sesaminol diglucoside increases. Beyond oil extraction, defatted sesame meal is a valuable by-product containing approximately 53.7% protein, 32.5% carbohydrates, 6.5% minerals, and significant amounts of lignan glycosides. This meal exhibits notable antioxidant properties primarily attributed to its phenolic compounds and lignan glycosides [3].

Scientific research has increasingly focused on exploring the potential therapeutic applications of these compounds. Sesamol, in particular, has attracted significant attention for its potential to prevent various diseases, including cancer, liver disorders, cardiac ailments, and neurological conditions [4,5,6,7]. Animal-level clinical studies, such as [8], have tried to address limited solubility, stability, bioavailability, and therapeutic efficacy by proposing innovative approaches such as specified delivery systems [8]. Such novel approaches represent a promising avenue for establishing sesamol as an efficient first-line treatment for various diseases.

Sesame oil cake, also referred to as sesame meal after grinding, is a by-product rich in functional compounds that has been overlooked in extraction research. Emerging technologies have shown promise in sustainable waste valorization and yielding value-added extracts [9]. In a study on applying such technologies for valorizing sesame processing by-products, ultrasound technology showed promise in extracting valuable compounds from sesame oil cake, surpassing traditional methods in yield, antioxidant activity, and lignan content, with the capability of producing large quantities of an extract rich in sesamol 1,2-diglucoside [3]. At the same time, the burgeoning consumer demand for healthy, eco-friendly bioactive compounds has driven significant advancements in other green extraction technologies, particularly supercritical liquid extraction. Recent studies [10,11] have highlighted these emerging technologies as clean processes capable of recovering valuable bioactive compounds with antioxidant, antibacterial, antiviral, and antifungal activities from diverse sources, including agricultural and marine food waste. Expanding on this perspective, Singh [12] explored high-value metabolites from micro- and macro-algae, demonstrating the potential of these green extraction technologies to address sustainability challenges and extract metabolites with significant biological activities, including antioxidant, anti-inflammatory, anticancer, and antimicrobial properties. Dashtian [13] further advanced the field by developing sophisticated frameworks integrating multiple extraction methodologies.

However, there is a lack of sustainable and efficient eco-friendly methods based on supercritical extraction to valorize sesame meal into high-value functional compounds combined with information on extracts’ quantitative bioactive content and neuroprotective properties. To bridge this gap, the present study aimed to develop a new methodology based on supercritical carbon dioxide extraction with ethanol as a co-solvent, enhance lignan extraction efficiency by process optimization, quantitatively analyze the extract, and assess the neuroprotective potential of the extracts under ischemic stress conditions.

## 2. Results

### 2.1. Investigation of Parameters for Supercritical Carbon Dioxide Extraction

Supercritical carbon dioxide (SC-CO_2_) extraction faces significant challenges due to carbon dioxide’s inherently low polarity, which reduces its effectiveness in extracting highly polar substances. To overcome this limitation, we investigated using co-solvents to enhance extraction efficiency. While nonpolar co-solvents like alkanes can improve the extraction of nonpolar hydrocarbons, our study focused on polar co-solvents, specifically water and ethanol, to assess their impact on sesame meal extract yield and antioxidant properties. Previous research by Qamar [14] showed that SC-CO_2_ preferentially extracts lower molecular weight and low-polarity compounds, while highly polar substances such as sugars and amino acids remain challenging to extract. Our study systematically investigated multiple extraction parameters, including temperature, pressure, CO_2_ volume, and ethanol concentration. We methodically varied ethanol concentration from 2 to 25 mol% to determine optimal extraction conditions. By pre-mixing ethanol with SC-CO_2_ and carefully controlling process variables, we aimed to overcome the inherent limitations of supercritical fluid extraction and maximize the extraction of polar compounds from sesame meal.

**Figure 1:**
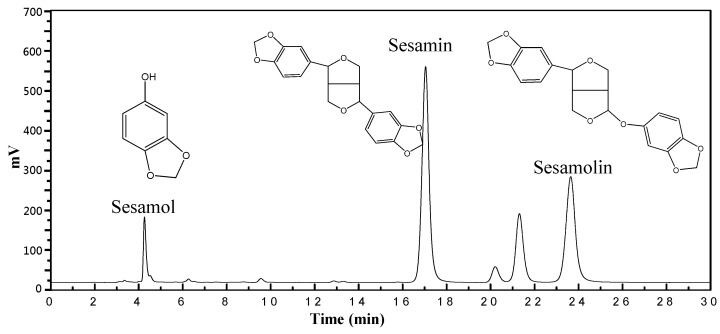
HPLC chromatograms of lignans from SC-CO_2_ at 6000 psi, 50 °C, 10 mol% EtOH, and 100 NL.

Our research revealed the complex interplay between various supercritical fluid extraction factors, including temperature, pressure, polarity, and extraction time. Adding ethanol as a co-solvent significantly enhanced the extraction of lignans from sesame meal using SC-CO_2_. Through careful optimization of extraction parameters, we demonstrated the possibility of achieving high extraction yields while preserving the bioactive properties of the extracted compounds. This comprehensive approach shows the potential of modified SC-CO_2_ extraction techniques for isolating valuable compounds from complex biological materials. Further research is needed to explore optimal conditions for different plant materials and investigate the potential of alternative co-solvents. As shown in Figure 1, the chromatogram displays the separation of neuroprotective compounds, including sesamol, sesamin, and sesamolin, extracted from sesame meal using supercritical carbon dioxide.

#### 2.1.1. Effect of Supercritical Carbon Dioxide Extraction Temperature on Sesame Lignans

**Figure 2:**
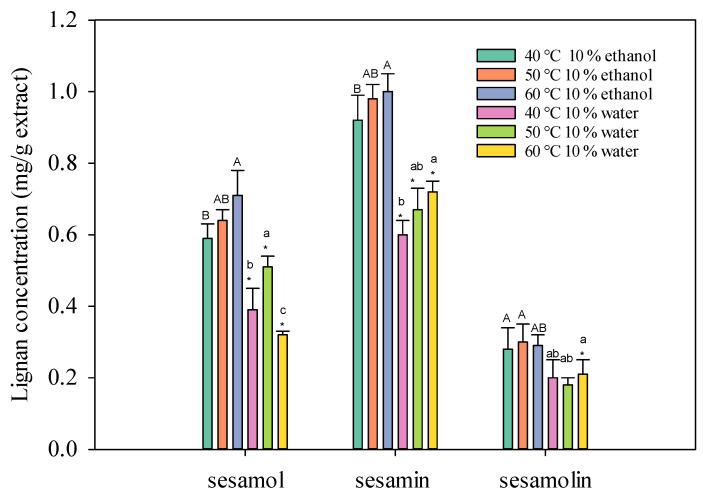
Effect of SC-CO_2_ temperature on the lignans concentration of sesame meal extracts (extraction conditions: 10 mol% EtOH, 100 NL, 4000 psi). Values are presented as means ± SD (*n* = 3). This means that the same co-solvent but different extracted temperatures, with different lowercase and uppercase letters, respectively, were significantly different based on Duncan’s multiple range test (*p* < 0.05). An asterisk (*) means significant (*p* < 0.05) differences between the same extracted temperature and different co-solvents assayed by a one-way ANOVA, followed by an unpaired Student’s *t*-test.

The results indicate that adding a co-solvent significantly influences the extraction efficiency of lignans from sesame meal using supercritical carbon dioxide. As a co-solvent, water proved ineffective in extracting oil-soluble substances due to its low miscibility with supercritical carbon dioxide. This limited miscibility resulted in subcritical conditions and incomplete mixing of the two phases, hindering the modification of fluid properties and the extraction process [15]. In contrast, as a co-solvent, ethanol demonstrated superior performance in enhancing the extraction of lignans. By altering the polarity and density of supercritical carbon dioxide, ethanol facilitated the solubilization and extraction of polar compounds such as sesamol and sesamin. As shown in Figure 2, it is essential to note that the extraction temperature plays a crucial role in the efficiency of the process. Exceeding 60 °C led to increased volatility of the solute and co-solvent, negatively impacting the extraction rate. Therefore, careful temperature control is essential to maximize the yield and quality of the extracted lignans.

The temperature of the SC-CO_2_ fluid also influences the extraction yield. In general, higher temperatures can increase the solubility of solutes in the SC-CO_2_ fluid, leading to higher extraction yields [16,17]. However, excessively high temperatures can degrade thermolabile compounds and reduce the density of the SC-CO_2_ fluid, which can negatively impact the extraction process.

#### 2.1.2. Effects of Different Co-Solvent Concentrations and Carbon Dioxide Consumption on the Supercritical Fluid Extraction of Sesame Lignans

Adding a co-solvent to supercritical carbon dioxide (SC-CO_2_), extraction significantly improved the extraction efficiency of oil-soluble antioxidants, such as lignans (sesamol and sesamin), from sesame meal compared to water. As a co-solvent, ethanol proved particularly effective in enhancing the extraction process. In previous studies, the enhanced polarity, density, and encapsulation of oil-soluble substances contributed to the improved extraction efficiency with ethanol [18,19]. Ethanol, being more polar than CO_2_, increases the polarity and density of the SC-CO_2_ fluid. This enhanced polarity allows for better solvation and extraction of polar compounds such as lignans. Ethanol interacts with oil-soluble substances in sesame meals and encapsulates them. When CO_2_ enters the extraction vessel, these encapsulated substances dissolve more readily into the SC-CO_2_ fluid, facilitating extraction. Higher temperatures can reduce the desorption energy required for solutes to detach from the sample matrix, making it easier for SC-CO_2_ to extract them.

**Figure 3:**
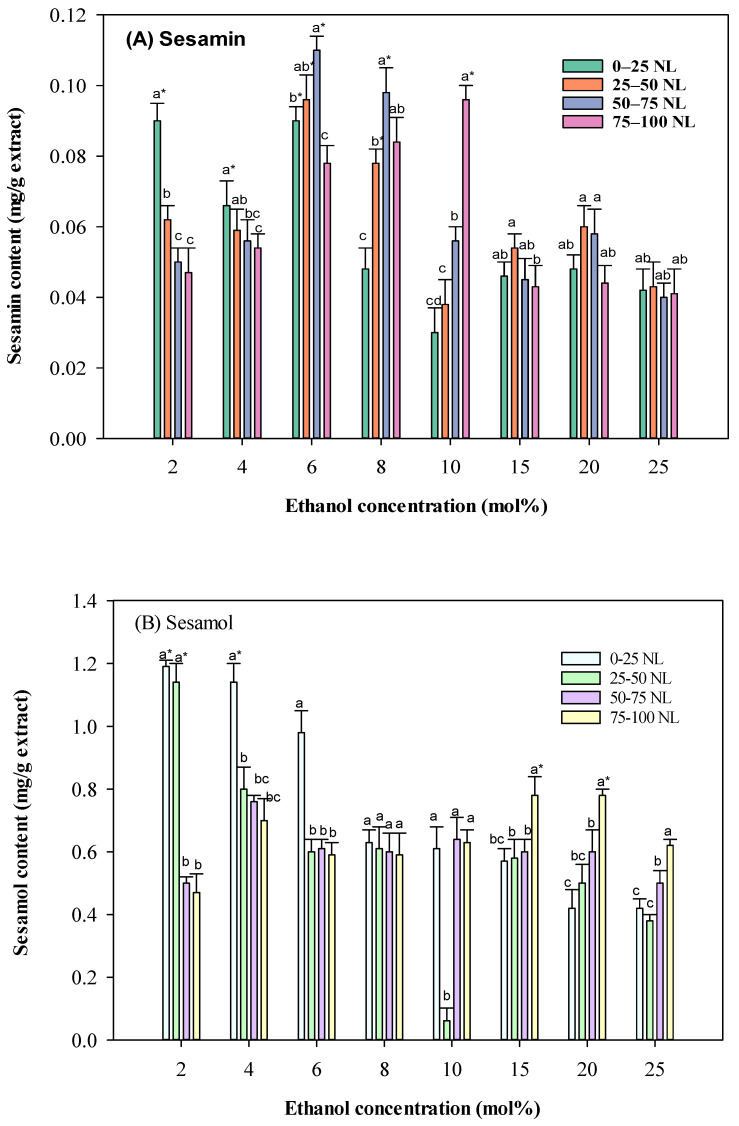
Effect of different ethanol concentrations on sesame meal extract’s sesamol and sesamin contents (extraction conditions: 4000 psi, 100 NL, 60 °C). Values are presented as means ± SD (*n* = 3). Means, for the same ethanol concentration but different CO_2_ consumption, with different lowercase letters, respectively, were significantly different based on Duncan’s multiple range test (*p* < 0.05). An asterisk (*) means significant (*p* < 0.05) differences between the same CO_2_ consumption and different ethanol concentrations assayed by a one-way ANOVA, followed by an unpaired Student’s *t*-test.

The influence of co-solvent concentration and CO_2_ volume: As shown in Figure 3, ethanol concentration as a co-solvent significantly impacted the extraction yield of lignans. Increasing the ethanol concentration from 2 to 10 mol% led to a higher extraction yield of sesamol. However, further increasing the ethanol concentration beyond 10 mol% did not significantly improve the yield. This is likely due to the competition between ethanol and SC-CO_2_ in extracting solutes. At higher ethanol concentrations, the increased polarity of the fluid may reduce its selectivity for specific compounds, leading to a decrease in the proportion of sesamol in the extract. In a recent study on solvent’s effects on protein extraction from sesame meal [20], eutectic solvents based on choline chloride-oxalic acid DES (deep eutectic solvent) were suggested to extract protein with desirable solubility. It should be mentioned that the present study targeted bioactive compounds with neuroprotective properties. Therefore, the optimal solvent identified is different from that of the abovementioned study.

The volume of CO_2_ also played a crucial role in the extraction process. Higher CO_2_ volumes generally led to higher extraction yields, especially at lower ethanol concentrations. This is because higher CO_2_ volumes provide a larger solvent volume for the extraction of solutes. However, at higher ethanol concentrations, the increased polarity of the fluid may reduce the effectiveness of CO_2_ as a solvent, leading to a less significant impact of CO_2_ volume on the extraction yield.

#### 2.1.3. The Influence of Pressure on SC-CO_2_ Extraction of Sesame Lignans Yield

**Figure 4:**
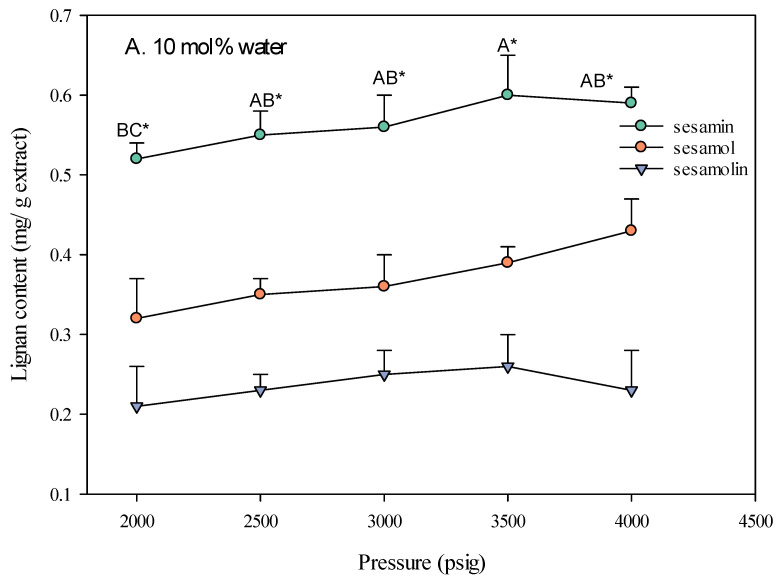

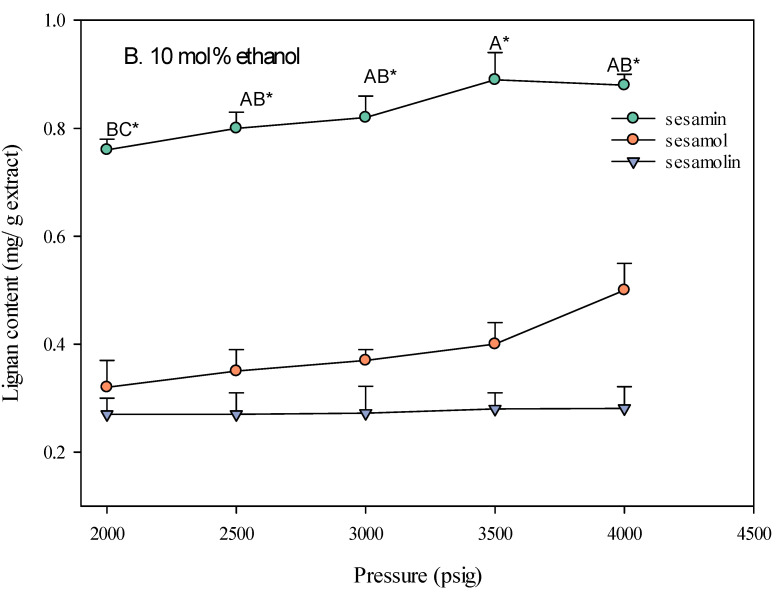
Effect of SC-CO_2_ pressure on the lignan content of sesame meal extracts (extraction conditions: 10 mol% EtOH, 100 NL, 60 °C). Values are presented as means ± SD (*n* = 3). Means, for the same lignin content but different extracted pressure, with different uppercase letters, respectively, were significantly different based on Duncan’s multiple range test (*p* < 0.05). An asterisk (*) means significant (*p* < 0.05) differences between the same extracted pressure and different lignin contents assayed by a one-way ANOVA, followed by an unpaired Student’s *t*-test.

The pressure of the supercritical carbon dioxide (SC-CO_2_) fluid significantly impacts the extraction yield of lignans from sesame meal. As shown in Figure 4, increasing the pressure generally led to a higher extraction yield, particularly when ethanol was used as a co-solvent. This is attributed to the increased density of the SC-CO_2_ fluid at higher pressures, which enhances its solvating power and allows for better extraction of solutes [21]. The type of co-solvent used also played a crucial role in the effect of pressure on extraction yield. The extraction yield increased with pressure when ethanol was used as a co-solvent. This is because ethanol, being more polar than CO_2_, can enhance the polarity and density of the SC-CO_2_ fluid [22], making it more effective in solvating and extracting polar compounds such as lignans. In contrast, when water was used as a co-solvent, the effect of pressure on the extraction yield was less pronounced. This is due to the limited miscibility of water with SC-CO_2_, which restricts water’s ability to modify the fluid’s properties significantly.

The lignan content of sesame meal extracts varied with pressure and the type of co-solvent used. When ethanol was used as a co-solvent, the sesamin content was highest at higher pressures (4000 psi), followed by sesamol and sesamolin. This suggests higher pressures are more effective in extracting lipophilic compounds such as sesamin. The optimal extraction conditions, identified as a pressure of 4000 psi, a temperature of 60 °C, and adding 10 mol% ethanol, resulted in the highest yield of these valuable bioactive compounds. This extract was used as a sample for subsequent bioactivity testing.

### 2.2. HPLC Results

Figure 1 presents the HPLC results, quantitatively identifying the lignan compounds extracted from sesame meal under supercritical fluid extraction conditions. These compounds include sesamin, sesamol, and sesamolin. The concentration of each compound varied significantly based on the specific extraction conditions. Notably, sesamin was the predominant compound, with concentrations ranging from 0.58 to 1.16 mg/g extract. Sesamol and sesamolin followed, with concentrations ranging from 0.38 to 0.81 mg/g extract and 0.17 to 0.37 mg/g extract, respectively. As reported in recent studies, the components mentioned above have significant biological effects [23,24]. A recent report highlighted the benefits of the bioactive compounds in sesame seeds [25]. Also, a previous report explained how sesaminol could be subjected to biotransformation in intestinal microflora [8]. Therefore, other beneficial components might also be presented in sesame mill extracts that could be assessed in future studies. Integrating such data with the results of the present study further highlights the importance of valorizing this agri-food waste for developing value-added products with commercial and health-promoting values.

### 2.3. Evaluation of the Bioactivity (LDH Release) of Supercritical Fluid Extraction of Sesame Lignans

**Figure 5:**
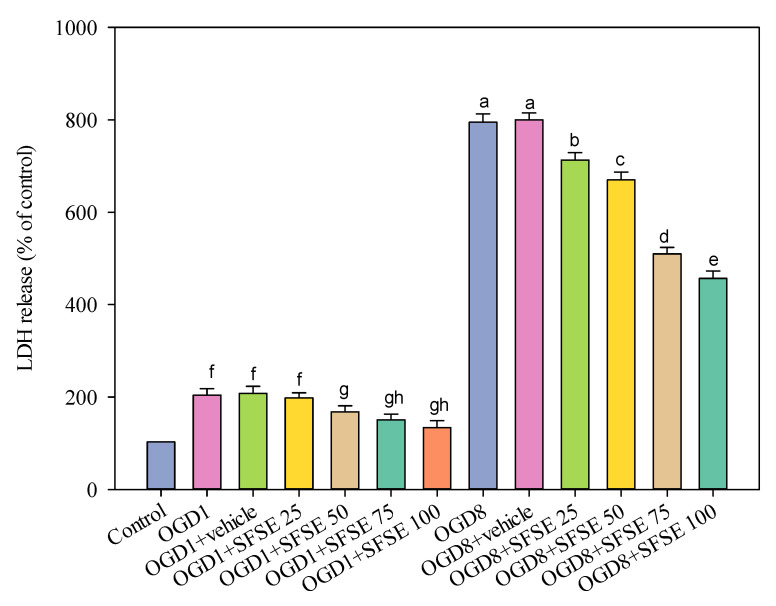
The effect of OGD1 (1 h), OGD8 (8 h), and SFSE (25, 50, 75, 100 μg/mL extract) on LDH release, indicating effective prevention of LDH release after 1 and 8 h OGD due to the pre-treatment with 50 μM of LY294002. Meaning, for the OGD test but treated with different SFSE concentrations, they are indicated by different lowercase letters. Data are presented as mean ± SEM of six independent experiments; OGD: oxygen–glucose deprivation, LDH: lactate dehydrogenase, LY: LY294002, SFSE: supercritical fluid sesame extract, 4000 psi, 100 NL, 10 mol% EtOH, 60 °C.

The study further explored the neuroprotective potential of the extracted lignans, leveraging in vitro cellular models to elucidate their mechanisms of action. Exposure of PC12 neural cells to oxygen–glucose deprivation (OGD) conditions, simulating ischemic stress, revealed the protective capabilities of supercritical fluid sesame extract (SFSE). The SC-CO_2_ extracts effectively mitigated cellular damage, as evidenced by the reduction in lactate dehydrogenase (LDH) release, a key marker of cell injury. Mechanistic investigations suggested that the neuroprotective effects were potentially mediated by the modulation of antioxidant defense systems [26]. Sesamol, a prominent lignan in the extract, demonstrated the ability to potentiate the activities of crucial antioxidant enzymes, such as superoxide dismutase and catalase, which play vital roles in neutralizing free radicals and safeguarding neural tissues against oxidative stress [27,28]. Further exploration using the PI3K/Akt pathway inhibitor LY294002 provided additional insights into the underlying cellular signaling mechanisms. Pre-treatment with LY294002 reversed the protective effects of the SFSE, indicating the involvement of specific signaling cascades in the observed neuroprotection. LDH release was significantly increased in PC12 cells exposed to 1 or 8 h OGD (Figure 5, *p* < 0.001 vs. control). SFSE (25 to 100 μg/mL) prevented the enhancement of LDH activity due to 1 h OGD (SFSE vs. OGD or OGD + vehicle group). SFSE reduced LDH release following 8 h OGD (SFSE vs. OGD or OGD + vehicle group). The supercritical fluid extract did not exhibit significant cytotoxicity under normal cellular conditions, suggesting its potential for safe and targeted therapeutic applications.

The findings of this study significantly contribute to the understanding of green extraction technologies and their applications in developing novel neuroprotective interventions. By demonstrating the efficacy of SC-CO_2_ extraction in isolating bioactive lignans and their subsequent protective capabilities against oxidative stress-induced neural damage, this study opens promising avenues for future neurological treatment strategies. The optimization of extraction methodologies and the elucidation of underlying neuroprotective mechanisms lay the foundation for developing advanced, sustainable, and targeted phytochemical-based interventions for neurological health.

### 2.4. Sustainability Impacts of the Results

Considering the worldwide concerns about environmental changes, global warming, and resource depletion, there is an urgent need to develop sustainable practices in the industry, and emerging food processing technologies hold significant promise in contributing to this critical shift [29]. In line with this, the capability of supercritical carbon dioxide extraction, as an emerging technology, was explored in this study, and the findings imply a significant sustainability impact aligning with the United Nations’ SDGs [29,30]. The methodology proposed in this study can contribute to achieving SDG 3 (Good Health and Well-being) by demonstrating the neuroprotective capacity of bioactive components obtained from sesame meal. Such capabilities can play roles in developing novel treatments based on natural compounds for oxidative stress-related neural injuries to enhance health outcomes. Also, the results support SDG 9 (Industry, Innovation, and Infrastructure) by optimizing processing parameters and providing innovation in applying novel food processing technology. The findings can also support SDG 12 (Responsible Consumption and Production) by promoting efficient use of resources and reducing waste through transforming sesame flour, a food processing waste, into value-added compounds. At the same time, the research can contribute to SDG 13 (Climate Action) by adopting a greener extraction technology with reduced environmental impact that can help reduce climate change’s impact.

## 3. Materials and Methods

### 3.1. Materials and Chemicals

#### 3.1.1. Reagents and Chemicals 

Ethanol (Taiwan Tobacco and Liquor Corporation, Taipei, Taiwan), methanol (100%, J.T. Baker, 9093-68, Phillipsburg, NJ, USA), glacial acetic acid (100%, J.T. Baker, 9508-03, Phillipsburg, NJ, USA), sesamol (Sigma, 128708, St. Louis, MO, USA), sesamin (Sigma, 9314, St. Louis, MO, USA), sesamolin: 97% purity, isolated and purified from sesame meal.

#### 3.1.2. Sesamolin Isolation and Structural Characterization

Sesamolin (97% purity, M.W. = 370; ESI^−^–MS, *m*/*z* 369.35 [M − H]^−^) was isolated from sesame meal using a modified extraction method. The sesame meal was stirred with hexane (1:10 g/mL) for 24 h. The extracted oil was then mixed with methanol (1:5 *v*/*v*), heated at 90 °C for 10 min, and stored at −20 °C for 24 h. The upper layer was collected and concentrated under reduced pressure. The crude extract was diluted with methanol (1:10 g/mL), filtered (0.45 μm membrane), and purified using preparative HPLC (Hyperprep 100 C18 column, 20 mm × 250 mm, 8 μm; Keystone, CO, USA) with methanol/H_2_O (7:3 *v*/*v*) as the mobile phase (flow rate: 5.0 mL/min, detection: 290 nm).

Previous studies [31,32] have shown that the stereochemical configuration of sesamin and sesamolin can be influenced by factors such as the extraction and analytical environment, often leading to an equilibrium mixture of enantiomers. To confirm the presence of the major component, (+) sesamolin, this study employed 1H-NMR spectroscopy with homo-decoupling analysis. The structure of isolated sesamolin was confirmed through ^1^H, ^13^C, HMBC, HSQC, and COSY NMR analyses using a Bruker Avance-500 MHz spectrometer (Bruker, Billerica, MA, USA, ^1^H: 500 MHz; ^13^C: 125 MHz) in CDCl_3_ with TMS as the internal standard at 25 °C. ^1^H NMR (500 MHz, CDCl_3_): δ = 2.88 (m, 1H, CH-8′), 3.14 (m, 1H, CH-8″), 3.87 (dd, *J* = 3.2, 8.9 Hz, 2H, CH-9′), 3.91 (dd, *J* = 2.7, 8.9 Hz, 2H, CH-9″), 4.33 (dd, *J* = 1.8, 7.2 Hz, 2H, CH-9′), 4.15 (dd, *J* = 6.6, 9.7 Hz, 2H, CH-9″), 4.75 (d, *J* = 3.3 Hz, 1H, CH-7″), 4.94 (d, *J* = 6 Hz, 1H, CH-7′), 5.88 (s, 2H, O-CH_2_-O), 5.94 (s, 2H, O-CH_2_-O), 6.43 (s, 1H, CH-3′), 6.49 (s, 1H, CH-6′), 6.78 (d, *J* = 10.5 Hz, 1H, CH-5″), 6.79 (dd, *J* = 1.4, 8.5 Hz, 1H, CH-6″), 6.85 (d, *J* = 1.4 Hz, 1H, CH-2”).

### 3.2. Supercritical Carbon Dioxide Extraction of Sesame Lignans

#### 3.2.1. Supercritical Carbon Dioxide Extraction System

**Figure 6:**
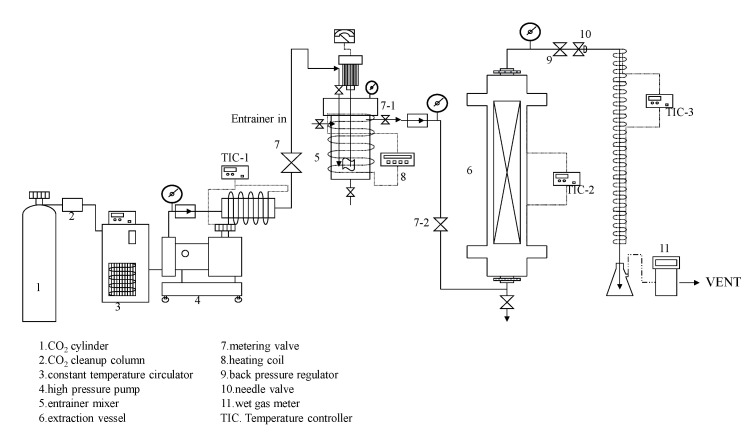
Schematic flow diagram of supercritical CO_2_ extraction for sesame meal.

The supercritical carbon dioxide (SC-CO_2_) extraction system is illustrated in Figure 6. The sample sesame seeds were placed into the extraction vessel (6). The operational procedure begins by activating a low-temperature thermostat pump (3) to cool the high-pressure pump (4), ensuring the CO_2_ remains liquid. The system is pressurized to the desired operating pressure, and the valve is opened to inject the supercritical fluid into the preheating chamber. Once the system pressure stabilizes, the high-pressure pump (4) maintains the set pressure, and the temperature in the preheating chamber (TIC-1, TIC-2) is controlled to reach the desired value. The valve (7-1, 7-2) is then opened to allow the fluid to flow into the extraction chamber, where the thermostat maintains the operational temperature. After the extraction chamber (6) pressure reaches the set point, the system is shut off to keep the operating pressure for 30 min. Subsequently, the needle valve (9, 10) is partially opened to allow the extracted fluid to flow into an ice-cooled Erlenmeyer flask. The flow rate and total volume are carefully controlled.

#### 3.2.2. Supercritical CO_2_ Extraction with Co-Solvent Method

For co-solvent extraction, the required amount of co-solvent is calculated based on the pre-determined CO_2_ volume. A specified co-solvent is placed into a stirred mixing chamber and agitated at 150 rpm. High-pressure CO_2_ is injected, and the system is pressurized to reach the supercritical state. During extraction, the pressure is maintained at the operational conditions, followed by a 30 min equilibration before dynamic extraction begins. Extraction pressure: 2000, 2500, 3000, 3500, and 4000 psi. Extraction temperatures: 40 °C, 50 °C, and 60 °C. Co-solvent concentration: 2–25 mol% ethanol. CO_2_ collection segments: 0–25, 25–50, 50–75, and 75–100 NL.

After collecting the solutes, the samples are left at room temperature to allow CO_2_ to dissipate and return to room conditions. The samples are then dried in an oven and weighed immediately upon removal, and the solute weight is calculated by subtracting the initial weight. The volume of CO_2_ passing through the Erlenmeyer flask is measured using a drum-type gas flow meter, which also provides the fluid temperature (via a thermometer) and atmospheric pressure (via a pressure gauge). Using reference data, the density (*ρ*) of CO_2_ is interpolated. The weight of CO_2_ is then calculated using the formula *ρ* × V = W. The solubility under specific operating conditions is determined as the ratio of sample weight to CO_2_ weight (sample weight [g]/CO_2_ weight [g]). Each measurement is repeated thrice, with reproducibility maintained within 5%.

### 3.3. High-Performance Liquid Chromatography (HPLC) of Lignans

The HPLC analysis was based on the method described by Kim [33]. Sesame meal SC-CO_2_ extract was dissolved in an n-hexane/chloroform mixture (2:1, *v/v*) and diluted to 10 mL to achieve a 0.1 g/mL concentration. The solution was filtered through a 0.45 μm membrane filter, and 20 μL of the filtrate was injected into the HPLC column. Column: Hypersil HS C18 (4.6 mm id × 250 mm, 5 μm particle size) from ThermoQuest, UK. The HPLC chromatographic mobile phase conditions for sesame lignans analysis are shown in Table 1. The mobile phase, a daily-prepared mixture of water and methanol, was degassed by vacuum filtration and sonicated for 20 min before use. A flow rate of 0.8 mL/min and a detection wavelength of 290 nm was used.

Purified standards at various concentrations were analyzed under the same HPLC conditions for sesamol, sesamin, and sesamolin analysis. The peak areas were used to generate standard calibration curves. By interpolation, the peak areas obtained from sesame oil samples under different roasting conditions were used to calculate the concentrations of sesamol, sesamin, and sesamolin.

### 3.4. Ischemic Stroke In Vitro Model and Treatments

This study established an in vitro ischemic stroke model using oxygen–glucose deprivation (OGD) in PC12 cells (BCRC 60048, supplied by FIRDI-BCRC (Food Industry Research and Development Institute-Bioresource Collection and Research Center, Hsinchu, Taiwan). The experimental procedure began by replacing the original culture medium with pre-warmed, glucose-free Hepes buffer supplemented with an antimycotic–antibiotic solution. Cells were then transferred to an anaerobic chamber flushed with 5% CO_2_ and 95% N_2_ and maintained at 37 °C for 1 and 8 h, with deoxygenation continuously monitored using oxygen-sensing probes. We prepared multiple treatment groups, including sesame supercritical fluid extraction extracts at varying concentrations of 25, 50, 75, and 100 μg/mL, optimized under extraction conditions of 4000 psi, 100 NL, 60 °C, and 10 mol% EtOH. Vehicle controls and blank nanocarriers were also included. Treatments were administered 24 h before and at the onset of OGD. When specific effects were observed, a PI3K inhibitor (LY294002) dissolved in dimethyl sulfoxide was applied at a 50 μM concentration 20 min before the effective agent [28]. Cell viability was assessed using the MTT colorimetric assay. A 20 μL MTT stock solution was added to each well 4 h before re-oxygenation. After aspirating the culture medium and dissolving formazan crystals in DMSO, absorbance was measured at 570 nm. Viability was calculated as a percentage of untreated control cells, with results expressed as mean ± standard error from six independent experiments. We measured lactate dehydrogenase (LDH) activity to quantify cellular damage, correlating with cell membrane integrity. Following re-oxygenation, media were removed, and cells were washed with PBS and then lysed with Triton X-100 to determine maximum LDH release. The percentage of LDH release was calculated by comparing LDH levels in the media to total LDH content, providing a comprehensive assessment of cellular damage in the OGD model.

### 3.5. Statistical Analysis

Data obtained from repeated measurements were expressed as mean ± standard deviation. Differences between experimental values were analyzed using Duncan’s new multiple range test, correlation analysis, and unpaired student’s *t*-test, all performed using the SAS (Statistical Analysis System, version 9.4) software package.

## 4. Conclusions

The findings of this comprehensive study highlight the significant potential of SC-CO_2_ extraction, particularly when combined with a suitable co-solvent like ethanol, as a promising technique for isolating bioactive compounds from plant materials. By carefully optimizing the extraction parameters, including the co-solvent concentration, CO_2_ volume, and temperature, we achieved high extraction yields while maintaining the quality and integrity of the extracted compounds. The core findings demonstrated the critical impact of ethanol as a co-solvent in enhancing the extraction efficiency of polar lignans, such as sesamol and sesamin, from sesame meal. Adding ethanol increased the polarity and density of the SC-CO_2_ fluid, enabling improved solvation and extraction of these target compounds. Furthermore, the co-solvent’s ability to encapsulate oil-soluble substances facilitated their dissolution into the supercritical fluid, streamlining the extraction process. Systematic analysis of extraction parameters revealed the delicate balance required to optimize the process. Increasing ethanol concentration from 2 to 10 mol% progressively enhanced the yield of sesamol, a key lignan of interest. However, elevating the ethanol concentration beyond 10 mol% resulted in diminishing returns, likely due to competitive interactions between the co-solvent and SC-CO_2_. The volume of CO_2_ and the extraction temperature also emerged as crucial factors, with higher CO_2_ volumes generally correlating with increased extraction yields, particularly at lower ethanol concentrations. Moderate temperature increases promoted higher yields, but excessively high temperatures posed the risk of degrading thermolabile compounds. These findings provide valuable insights for developing efficient and sustainable extraction processes for bioactive compounds from plant materials. The optimization of extraction methodologies, combined with the elucidation of underlying neuroprotective mechanisms, lays the foundation for advancing green extraction technologies and their application in developing novel phytochemical-based interventions for neurological health.

## Figures and Tables

**Table 1:** HPLC chromatographic mobile phase conditions.

Time (min)	Mobile Phase Ratio (%)	
	A [Pure Water]	B [Methanol: Pure Water (8:2)]
0	90	10
6.0	90	10
7.0	85	15
10.0	85	15
25.0	50	50
26	25	75
35	25	75

## Data Availability

Data are available on request from the authors.

## Abbreviations

SC-CO_2_: supercritical carbon dioxide; CO_2_: carbon dioxide; SFSE: supercritical fluid sesame extracts.
